# Pretreatment clamping of pulmonary artery during uniportal thoracoscopic lobectomy

**DOI:** 10.1186/s12893-020-00826-4

**Published:** 2020-07-22

**Authors:** Ruijie Zhang, Yixin Cai, Tiffany Wang, Xiangning Fu, Ni Zhang

**Affiliations:** 1 Department of Thoracic Surgery, Tongji Hospital, Tongji Medical College, Huazhong University of Science and Technology, Wuhan, 430030 P.R. China; 2 University of Maryland School of Medicine, Baltimore, MD 21201 USA

**Keywords:** Uniportal VATS, Pretreatment clamping of pulmonary artery, Pulmonary artery bleeding, Conversion rate

## Abstract

**Background:**

Intraoperative pulmonary artery (PA) hemorrhage is one of the leading reasons for conversion from uniportal VATS to open thoracotomy, especially for the small incision (≤3 cm) uniportal VATS performed by our department. So, We designed a technology called pretreatment clamping of the pulmonary artery, which may be helpful to solve the problem.

**Methods:**

A retrospective analysis of 19 patients who had pulmonary artery bleeding during uniportal thoracoscopic lobectomy in which one group had undergone preventive pulmonary artery clamping, the clamping group (*n* = 11), and one group which did not receive preventive clamping, the non-clamping group (*n* = 8). We compared the rates of conversion from the uniportal VATS approach to open thoracotomy or multi-incision operation, duration of pulmonary artery repair, blood loss, length of postoperative hospital stay and postoperative complications of the two groups.

**Results:**

Compared to the non-clamping group, the clamping group had lower rates of conversion to open thoracotomy (0% vs 62.5%, *p* < 0.05) and lower rates of conversion to multi-incision operations (18.2% of non-clamping converted to 2-port approach vs 12.5% of clamping converted to 2-port approach and 12.5% converted to 3-port approach, *p* < 0.05). Duration of pulmonary artery repair was reduced in the clamping group (10.1 ± 3.2 min vs 18.3 ± 5.5 min, *p* < 0.05). The clamping group also had decreased blood loss (23.6 ± 11.2 ml vs 47.5 ± 14.9 ml, *p*<0.05). There were no significant differences in postoperative hospital stay and postoperative complications between the two groups.

**Conclusion:**

Pretreatment clamping of the pulmonary artery in VATS lobectomy can decrease conversion rates, decrease blood loss, shorten repairing time of the pulmonary artery, and feasibly can be applied in uniportal thoracoscopic lobectomy.

## Background

Video-assisted thoracic surgery (VATS) is a technique in minimally-invasive surgery that has developed quickly in recent decades, which provides similar long-term survival outcomes as open thoracotomy, but with less postoperative pain and a shorter recovery time [1–3]. Classically, the approach for VATS has been through 3 or 4 ports, but recently, the number of incision sites has been reduced to one, called uniportal VATS or single incision thoracoscopic surgery (SITS) [1–3]. Intraoperative pulmonary artery (PA) hemorrhage is one of the leading reasons for conversion from uniportal VATS to open thoracotomy [4], especially for the small incision (≤3 cm) uniportal VATS performed by our department [5]. The single small incision presents difficulties for pulmonary artery clamping because quickly inserting the auricle clamps to occlude the pulmonary artery results in a blurred planar field of vision [6, 7]. However, even if the auricle clamps are inserted in time, there is generally no more space for instrumentation for pulmonary artery repair. Thus, pulmonary artery bleeding usually leads to an increase in the number of operation incisions or conversion to open thoracotomy. In this long-term study by our department, selective preventive PA clamping was performed on patients at high risk for PA hemorrhage. According to our findings, this method can increase the success and safety of pulmonary artery repair in uniportal VATS and make adding operation incisions and conversion to open thoracotomy unnecessary, sparing patients longer operation times, hospital stays, and increased postoperative pain.

## Methods

This study was Retrospective study and consent was obtained from each patient before surgery and data collection. Informed consent was obtained from all individual participants involved in the study.

### Patients data

We retrospectively analyzed 19 patients who suffered pulmonary arterial hemorrhage during small-incision thoracic lobectomy at our institution from September 2014 to December 2016. Preventive PA clamping technique was performed if preoperative CT indicated that tumor,10(hilar) and/or 11(interlobar) lymph node might adhere to the PA and might cause PA rupture during surgery. In the clamping group, preventive pulmonary artery clamping was performed and in the non-clamping group, preventive PA clamping was not performed. Patient data of the two groups is shown in Table 1.

**Table 1:** General information of 19 patients

Category		Clamping group (11 cases)	Non-clamping group (8 cases)
Gender	Male	8	6
	Female	3	2
Median age (years)		52	54
Resection site	Right upper lobe	5	4
	Right lower lobe	2	1
	Left upper lobe	3	2
	Left lower lobe	1	1
Cause of bleeding	Lymph node incarceration	9	7
	Electrocoagulation hook accidental injury	2	1

### Uniportal VATS

A single small incision (3 cm) was made in the 5th intercostal space at the midaxillary line on the side of the operation (on the left side the 6th intercostal space can also be selected). We introduced the thoracoscope into the port and performed the modular lung cancer radical operation [5]. The right side is based on the lower mediastinal module-the subcarinal module-the anatomical lobectomy-the upper mediastinum module, and the left side is based on the inferior mediastinal module-the subcarinal module-the upper mediastinal module-anatomical lung resection. The hilum and pulmonary artery were thoroughly dissected and exposed. All the Uniportal VATS here were performed by the same surgeon, Dr. Ni Zhang.

### Pulmonary artery repair

In each case, pulmonary artery hemorrhage was repaired using a 4–0 prolene suture for continuous valgus suture, then the artery lumen was flushed with heparin and normal saline. The clamped pulmonary artery was vented before the last needle was tied. Ten patients total completed their procedures using the small single-incision VATS approach. In 3 cases one incision was added, in 1 case two incisions were added, and 5 cases were converted to open thoracotomy.

### The preventive pulmonary artery clamping technique

**Fig. 1:**
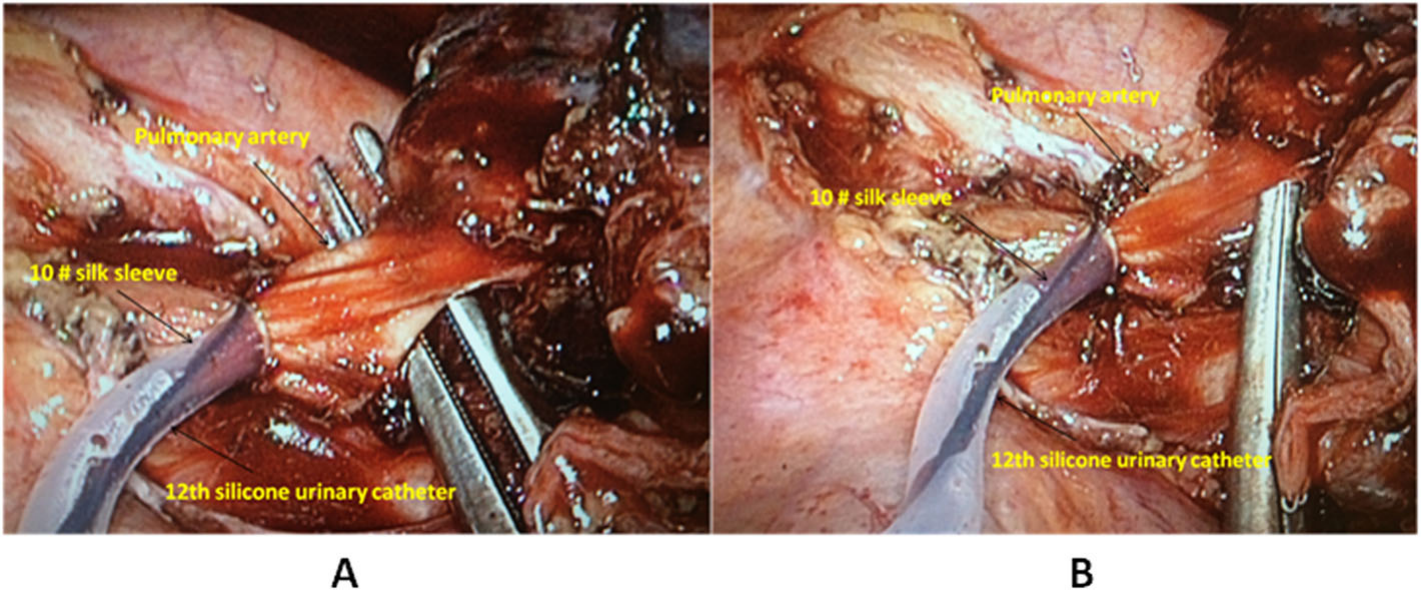
Preventive pulmonary artery blocking technique: **a** separation of the pulmonary artery (left); **b** Pulmonary artery separation completed (right)

The preventive pulmonary artery clamping technique is shown as Fig. 1.

12-French silicone urinary catheter was trimmed to the length from the incision to the pulmonary artery. Wrap around the pulmonary artery with a 10 # silk sleeve, then pull the two tips of the sleeve out through the ureteral cavity. Clamp the ends of the sleeve segments with mosquito clamp. If intraoperative pulmonary artery bleeding occurs, immediately pull the silk thread and push the urinary catheter to the opposite direction. Thus, the pulmonary artery was clamped, facilitating uniportal VATS pulmonary artery repair.

### Peri-operative management

Low molecular weight heparin was injected subcutaneously daily beginning 24 h after surgery if patients did not have contraindications. Early mobilization was encouraged 2 days post-op. Patients were administered anti-inflammatory and analgesic treatment for pain and symptom management. Patients were also instructed on deep breathing exercises and given atomization treatment and assisted coughing to facilitate expectoration. Sputum aspiration via bedside fiberoptic bronchoscopy was performed as needed, along with daily observation of chest tube drainage and vital signs.

### Outcome measurements

Incidence of PA perforation or opening, duration of pulmonary artery repair (from onset of bleeding to completion of repair), blood loss during repair, duration of postoperative hospital stay and postoperative complications were evaluated.

### Statistical methods

Data was analyzed using SPSS 19.0 software (SPSS Inc., Chicago, IL, USA). The chi-square test was used to assess conversion rate and complication rate. The independent-samples t-test was used to analyze duration of pulmonary artery repair, volume of blood loss, and length of postoperative hospital stay. Data are presented as the mean ± standard deviation (SD), with *p*-values < 0.05 considered significant.

## Results

### Conversion rate

In the clamping group, 9 cases underwent single-port thoracoscopic pulmonary angioplasty, in which 2 cases increased to two-incision VATS. There were no conversions to open surgery in the clamping group and the conversion rate to two-port surgery was 18.2%.

There were 8 cases in the non-clamping group. After PA bleeding in one case, the PA was clamped with thoracic forceps, then a uniportal VATS pulmonary artery repair operation was performed. In another case, the incision number was increased to two ports and the PA bleeding was also managed with thoracic forceps. Another case was increased to a three-port operation and the PA was clamped with auricle clamps to perform the repair surgery. In 5 cases, the operations were converted to open thoracotomy. The conversion rate to open thoracotomy was 62.5% and the conversion rate to two-incision and three-incision operations was 18.2%.

The conversion rates to open thoracotomy and multiple incision operation were significantly lower in the clamping group than the non-clamping group (*p* < 0.05) (Table 2).

**Table 2:** Technique results comparison of two groups

Category	Clamping group (11 cases)	Non- Clamping group (8 cases)	*P* value
Conversion (cases)			
No conversion	9	1	<0.05
Conversion to 2 incisions	2	1	
Conversion to 3 incisions	0	1	
Conversion to open thoracotomy	0	5	
Pulmonary artery repair time (min)	10.1 ± 3.2	18.3 ± 5.5	0.001
Blood loss (mL)	23.6 ± 11.2	47.5 ± 14.9	0.001
Postoperative hospital stay (days)	10.2 ± 1.6	10.4 ± 1.2	0.77

### Pulmonary artery repair time

The pulmonary artery repair time (the duration of time from bleeding to repair) was significantly shorter in the clamping group than in the non-blocking group (10.1 ± 3.2 min vs 18.3 ± 5.5 min, *P* < 0.05). The duration of time for pulmonary artery clamping in both groups was less than 30 min (Table 2).

### Blood loss

Blood loss in the clamping group was significantly less than that of the non-blocking group (23.6 ± 11.2 mL vs 47.5 ± 14.9 mL, *P* < 0.05) (Table 2).

### Postoperative hospital stay

There was no significant difference in the average length of postoperative hospital stay between the clamping group and non-clamping group (10.2 ± 1.6 days vs 10.4 ± 1.2 days) (Table 2).

### Postoperative complications

There were no deaths in either of the two groups after the operation and there were no serious complications such as massive hemorrhage, bronchopleural fistula, pulmonary artery stenosis, hemorrhage and pulmonary artery thrombosis. There were 6 cases of respiratory infection, 3 cases of arrhythmia and 2 cases of pleural effusion, all of which were managed with conservative treatment. There was no significant difference in complication rates between the clamping and non-clamping groups.

## Discussion

The first use of uniportal VATS for pulmonary nodule biopsy was reported in 2004 by Rocco [1]. With technological advancement in recent years, the scope of application of uniportal VATS has gradually expanded. Since then, the first lobectomy using uniportal VATS was reported in 2011 by Gonzalez-Rivas [8]. Subsequently, the uniportal VATS approach has been comprehensively developed and applied for lobectomy and segmentectomy. Increasing numbers of reports have confirmed that uniportal VATS is effective in reducing postoperative incision pain and paresthesias in patients, with equal outcomes compared to multi-port thoracoscopy and open thoracotomy [1–3, 9–11]. Uniportal VATS has been widely used in various kinds of thoracoscopic pulmonary surgery throughout many institutions and has replaced most multi-port thoracoscopic surgery [12, 13].

Some intraoperative complications can lead to the conversion from uniportal VATS to multi-port VATS or open thoracotomy, which can lead to increased morbidity, increased surgical time and length of hospital stay [14, 15]. Common reasons for conversion include pleural adhesions, aggressive tumor or lymph node invasion, requirement for bronchoplasty or other special surgical methods, pre-operative ventilatory failure, and pulmonary hemorrhage. Pulmonary artery hemorrhage is a dangerous intraoperative complication that is difficult to correct, making it one of the most significant challenges for thoracoscopic lobectomy [16–19].

Pulmonary artery hemorrhage is more likely to occur and more difficult to manage if it occurs in the uniportal VATS approach compared to open thoracotomy. In addition to the pulmonary artery’s increased susceptibility to injury due to its large size, there are several reasons for the increased risk of pulmonary artery hemorrhage in uniportal VATS. The uniportal VATS approach has a limited operating plane with less stereoscopic vision than in multi-port thoracoscopy and open thoracotomy. In addition, the electrocoagulation hook lacks effective haptic feedback, making control of depth and force difficult in the process of pulmonary artery separation. Also, visualization of the surgical field in uniportal VATS is more susceptible to occlusion by lung tissue and instrumentation.

These factors contribute to the increased risk of pulmonary artery bleeding during uniportal VATS, which may lead to unplanned conversion to open thoracotomy. Yet, if the pulmonary artery can be directly repaired in the uniportal VATS approach, conversion to a more invasive surgical approach may not be necessary. However, the actual operation is very difficult for several reasons. Firstly, in the uniportal VATS approach, especially the small ≤3 cm incision uniportal VATS carried out by our department, the incision size greatly limited the ability to quickly insert the auricle clamps and clamp the pulmonary artery. In addition, the bleeding results in the blurring of the plane field of vision which was already relatively limited. Secondly, even after adjusting the position of the forceps, there is generally extremely limited space for instrumentation for pulmonary artery repair, thus prolonging operation time and frequently leading to an increase in operation incisions or a conversion to open thoracotomy. Thirdly, the duration of time needed to suture the PA in the thoracoscopic approach exceeds the 30 min limitation on the duration of pulmonary artery clamping, which may lead to ischemia reperfusion injury, pulmonary edema, and other adverse events.

In the event of pulmonary artery bleeding, uniportal VATS lobectomy requires adequate time and space for instrumentation for pulmonary artery repair, rapid occlusion of the artery in order to keep the field of vision as clear as possible, and prevention of serious pulmonary artery hemorrhage. Besides constantly improving surgeon skill and experience, there remains a need for pulmonary artery clamping techniques that are more suitable for uniportal VATS. Thus, we have developed this preventive pulmonary artery clamping technique, with clear advantages for uniportal VATS. This clamping technique occupies very minimal space with simple instrumentation [8–10], providing sufficient operating space for pulmonary artery repair and minimizes instrumentation interference. In addition, preventive clamping can reduce pulmonary artery pressure, which may facilitate lymph node dissection—for lymph nodes or tumors with severe adhesions to blood vessels, pulmonary artery clamping can facilitate a difficult dissection [20, 21]. Also, the reduced tension in the distal pulmonary artery greatly lowers the risk of pulmonary artery rupture during dissection [20, 21]. Even if the pulmonary artery ruptures during dissection, no major bleeding will occur, and the repair can be unhurried. Moreover, preventive pulmonary artery clamping rapidly and effectively reduces bleeding, ensures a clear operating field, avoids accidental injury, and makes uniportal VATS easier to perform. The preventive pulmonary artery clamping technique is also relatively accessible for widespread implementation because it is easy to learn and perform and requires simple and common materials and skills.

According to our institution’s experience, in uniportal VATS, the preventative pulmonary artery blocking technique significantly reduced the operation conversion rate, blood loss and blood transfusion volume, and duration of pulmonary artery repair.

We propose several suggestions for the implementation of this technique:

1. This method is more suitable for cases at high risk for bleeding, such as tumor or lymph node adhesion to blood vessels. In conventional patients, pretreatment PA clamping may increase operation time and risk.
2. The surgeon must be skilled in thoracoscopic surgery, especially in the dissection of the pulmonary artery and vascular repair in the event that it becomes necessary.
3. Pretreatment PA clamping is more suitable for uniportal VATS, especially conferring more obvious advantages in small incision uniportal VATS, though the technique can certainly also be applied to multi-portal thoracoscopy and open thoracotomy.

In addition, our department has adhered to a “lymph node dissection first, pulmonary lobectomy later” protocol for about a decade whether for open thoracotomy or uniportal VATS [4, 5]. This protocol has several advantages: thorough dissection of the pulmonary hilum makes the blood vessels well-exposed and easy to manipulate. Also, if bleeding occurs, it is easier to get better visual field of operation through pulmonary lobes tugging. Additionally, pulmonary artery repair is easier without view obstruction by lymph nodes. Procedural operation is also easy to learn, which helps to shorten the learning curve for the uniportal VATS approach [5].

In conclusion, this preventive pulmonary artery clamping technique maintains space for instrumentation and provides effective occlusion of the PA. The technique is especially suitable for small (≤3 cm) single-incision thoracoscopic surgery in order to reduce the operation conversion rate caused by pulmonary artery hemorrhage. Due to a limited number of cases, more experience is needed before further promotion and application. However, preventive pulmonary artery clamping has the potential to make small-incision uniportal VATS procedures safer and smoother for patients.

**Additional file 1: Video legends.** Preventive pulmonary artery blocking technique. 12-French silicone urinary catheter was trimmed to the length from the incision to the pulmonary artery. Wrap around the pulmonary artery with a 10 # silk sleeve, then pull the two tips of the sleeve out through the ureteral cavity. Clamp the ends of the sleeve segments with mosquito clamp. If intraoperative pulmonary artery bleeding occurs, immediately pull the silk thread and push the urinary catheter to the opposite direction. Thus, the pulmonary artery was clamped, facilitating uniportal VATS pulmonary artery repair. Loose the sleeve after lymph node dissection.

## Data Availability

All data generated or analysed during this study are included in this published article and its supplementary information files.

## Abbreviations

VATS: Video-assisted thoracic surgery
SITS: Single incision thoracoscopic surgery
PA: Pulmonary artery
SD: Standard deviation
